# Orthodontic and Orthognathic Surgical Treatment of a Pediatric OSA Patient

**DOI:** 10.1155/2016/5473580

**Published:** 2016-08-10

**Authors:** Gregory W. Jackson

**Affiliations:** Department of Orthodontics (M/C 841), College of Dentistry, University of Illinois at Chicago, 801 S. Paulina Street, Chicago, IL 60612, USA

## Abstract

A case report is presented which demonstrates the effectiveness of comprehensive orthodontic treatment combined with orthognathic surgery in the correction of malocclusion and reduction in the sequelae of Obstructive Sleep Apnea (OSA). The patient's severe OSA was improved to very mild as evaluated by full overnight polysomnogram. The orthodontic treatment included the expansion of both dental arches and mandibular advancement surgery. There was significant improvement in the patient's sleep continuity and architecture with the elimination of obstructive apneas.

## 1. Introduction

Obstructive Sleep Apnea (OSA) is a serious and common disease in children which if untreated can lead to serious negative consequences including poor school performance, behavior and cognitive problems, heart disease, hypertension, and metabolic problems [1–3]. Sleep apnea is a disease characterized by recurrent episodes of breathing pauses (apneas) or decreases in airflow (hypopneas) during sleep resulting in arousals, fragmented sleep, and disturbance in normal sleep architecture [4]. Apneic events result in hypoxemia (low oxygen in the blood), hypoxia (low oxygen in the tissues), and hypercapnia (increased blood carbon dioxide). Patients may experience 30 to 300 events of greater than 10-second breathing pauses per night. Because of children's different physiology clinically relevant apneas may not last this long. Apneas of three to four seconds' duration can be accompanied by blood oxygen desaturations in children. However, children are more likely to have hypopneas than clear-cut apneas [5–8]. There are two types of sleep apnea: central sleep apnea and obstructive sleep apnea.

Obstructive apneas are characterized by an absence of oral and nasal airflow despite persistent inspiratory efforts [7]. OSA affects approximately 24% of men and 9% of women [9]. In children, OSA prevalence is 10% equally distributed between males and females [10–12]. More recently the prevalence of childhood obesity has gone up to 60%. This has led to an increase in awareness of pediatric sleep disorders such as obesity-related OSA. Timely diagnosis and management of pediatric OSA may prevent associated comorbidities [1, 12].

The primary medical surgical treatment of pediatric OSA is adenotonsillectomy (AT). Treatment of OSA with AT resulted in improvements in behavior and attention and likely improvement in cognitive abilities [10]. Quality of life in children with OSA has been shown to improve after AT [13]. Obese children, positive family history of OSA, and African American children are at high risk for having residual OSA after AT [14]. Abnormal mandibular development and malocclusion can affect the respiratory function of a patient. Children with habitual snoring and OSA have a unique craniofacial morphology [15, 16]. The craniofacial abnormality that leads to OSA may involve delayed growth of the mandible, leading to mandibular retroposition commonly found in patients with OSA. Mandibular retroposition is also associated with posterior displacement of the tongue [17]. This narrows the upper airway, predisposing it to collapse and contributing to the development of OSA [18]. Another common abnormality in patients with OSA is a narrow, high-arched palate. Orthodontic treatment can result in changes in both dental and maxillomandibular alignments and the airway which can help resolve OSA. A study of treatment of OSA with a rapid palatal expander (RPE) reported that in 9 of 10 patients maxillary expansion reduced symptoms [19]. A RPE was shown to be an effective treatment for OSA in children with enlarged tonsils and adenoids and also in treating adult OSA [20]. Several other studies have demonstrated the efficacy of RPE in treating pediatric OSA [21–24].

## 2. Case Report

**Figure 1:**
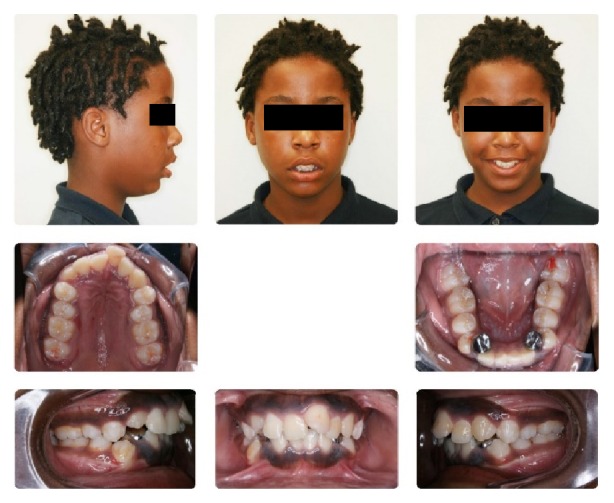
Initial composite.

**Figure 2:**
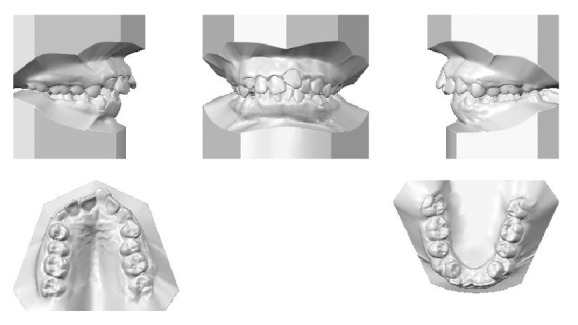
Initial models.

**Figure 3:**
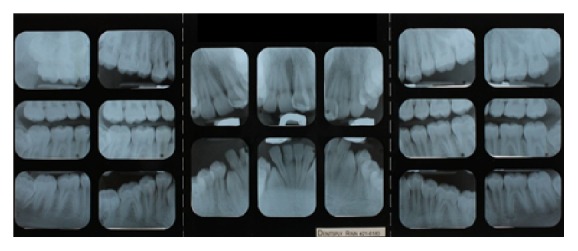
Initial full mouth radiographs.

**Figure 4:**
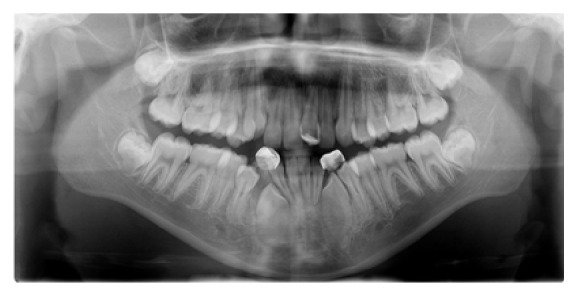
Initial panoramic radiograph.

**Figure 5:**
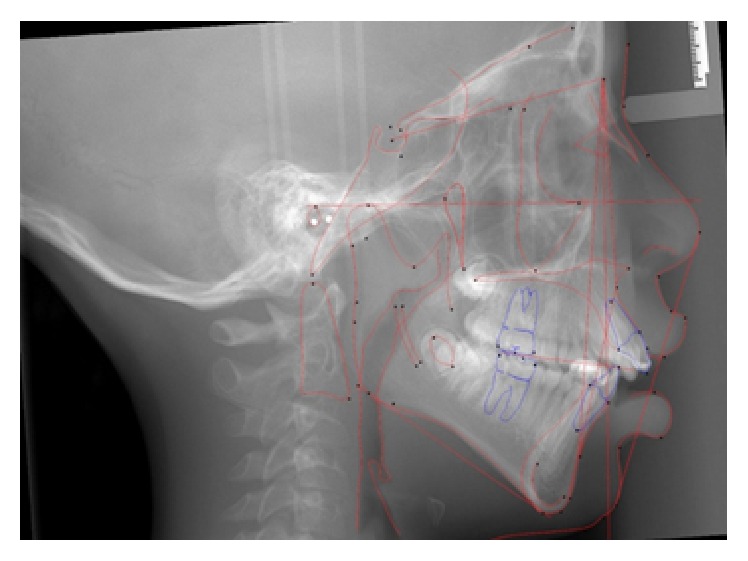
Initial cephalometric radiograph.

**Figure 6:**
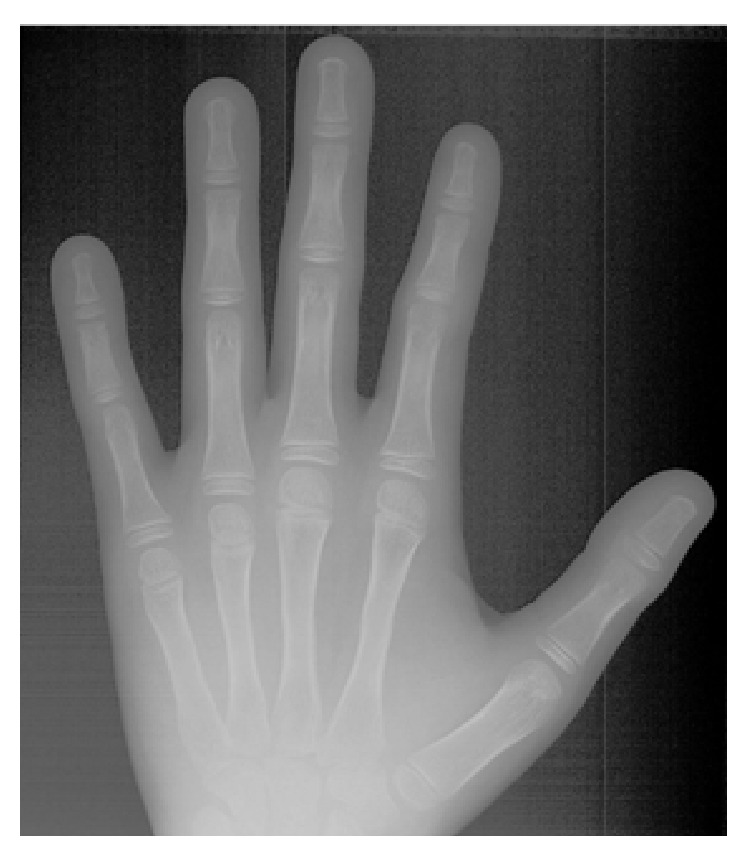
Initial hand-wrist radiograph.

**Figure 7:**
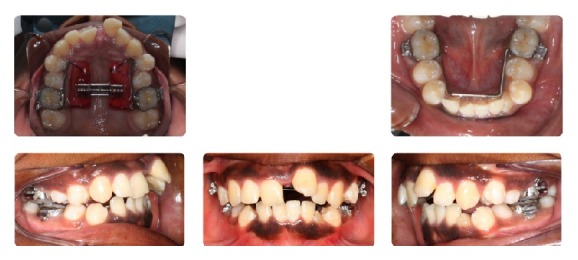
Progress photographs.

**Figure 8:**
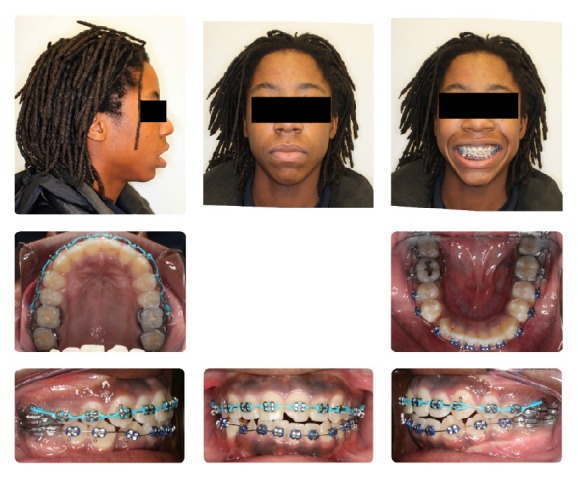
Progress photographs.

An 11-year-and-11-month-old African American male was being evaluated in the Pediatric Sleep Disorders Program at the University of Chicago for Obstructive Sleep Apnea. This diagnosis was confirmed by a sleep study performed on 4/10/2008. He was noted to have unrefreshing sleep, loud snoring, observed apneas, morning headaches, and difficulty waking from sleep. His sleep architecture was fragmented with microarousals due to respiratory events. The microarousal index was 19, which was increased. This study documented 14 obstructive apneas, no central apneas, 6 mixed apneas, and 81 hypopneas (of which 16 were associated with a 3% oxygen desaturation) over the 384 minutes of recorded sleep. The overall Apnea-Hypopnea Index (AHI) was 15.8. The non-REM AHI was 8 and the REM AHI was 72. The supine AHI was 17. The average oxyhemoglobin saturation was 98% while awake, 98% during REM, and 96% during non-REM sleep. The overall desaturation index during sleep was 4. The lowest oxygen saturation during sleep was 92%. The electrocardiogram (EEG) documented normal sinus rhythm. His average heart rate was 81 bpm and periodic limb movements were not observed. He was diagnosed with severe Obstructive Sleep Apnea which was worse during REM sleep. He was not considered to be a candidate for adenotonsillectomy by the Sleep Center and had difficulty tolerating a Continuous Positive Airway Pressure (CPAP) machine. He was found to have “significant orthodontic problems” and was referred to the University of Illinois Department of Orthodontics for screening. The patient's chief complaint was “I would like straighter teeth.” The patient had a history of sleep apnea, asthma, and ADHD. He was taking the following medications: Albuterol® and Flovent® for asthma and Concerta® for his ADHD. He had a symmetrical, mesofacial face and a retrognathic soft tissue profile (Figure 1). His lips were protruded and apart at rest with a high upper lip line. He had a Class I malocclusion in the late mixed dentition. Overjet was moderate at 4 mm. and overbite was deep also at 4 mm. and 50%. The maxillary arch was narrow and tapered with moderate crowding and the mandibular arch was narrow and ovoid with severe crowding having both permanent canines impacted and retained primary first molars with stainless steel crowns (Figures 3 and 4). There was a history of trauma to his upper left central incisor with no pulpal damage and a fractured mesial incisal edge (Figures 2 and 3). There was generalized hypocalcification and generally poor oral hygiene. The maxillary dental midline was 2 mm. left of his facial midline and the mandibular midline was centered. No occlusal cant was noted and the TMJs were normal. The cephalometric analysis showed a relatively well-positioned maxilla, retrusive mandible with an ANB angle of 7.3°, a high mandibular plane angle of 35.0°, convex skeletal and soft tissue profiles, an acute nasolabial angle, protrusive upper and lower lips, retroclined lower incisors, and cervical development of CVS 2 (Figure 5). Significant growth was also anticipated in this patient during treatment as shown in his hand-wrist radiograph (Figure 6). His treatment objectives were to improve airway capacity and help his OSA by expanding the upper and lower arches, resolving upper and lower crowding, correcting midlines, and proclination of lower incisors. Rapid maxillary expansion has been shown to be effective in treating children with OSA [25]. The patient was also informed of the possible need for mandibular advancement and/or advancement genioplasty if this orthodontic treatment alone did not reduce his OSA symptoms sufficiently. He underwent his first stage of comprehensive orthodontic treatment nonextraction with fixed edgewise appliances and a Haas palatal expander and mandibular Arnold expander (Figures 7 and 8). The patient was instructed to activate the Haas expander 2 turns or 1/2 mm. per day. After maxillary expansion the patient's mother reported the patient snoring less and not as loud with an absence of loud apneic periods. The patient also reported feeling more rested and having more energy.

**Figure 9:**
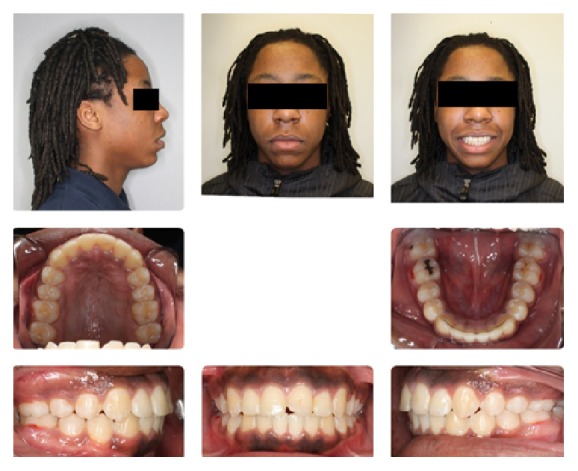
Stage 1: Orthodontic Treatment Final Composite.

**Figure 10 fig10:**
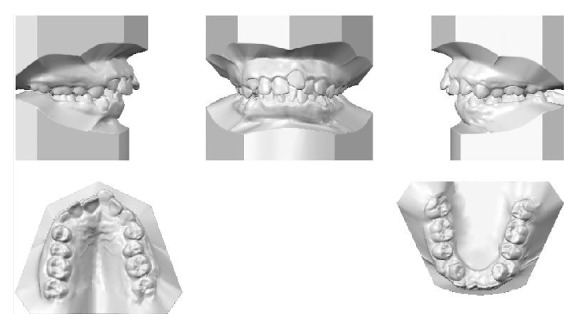
Stage 1: Orthodontic Treatment Final Models.

**Figure 11 fig11:**
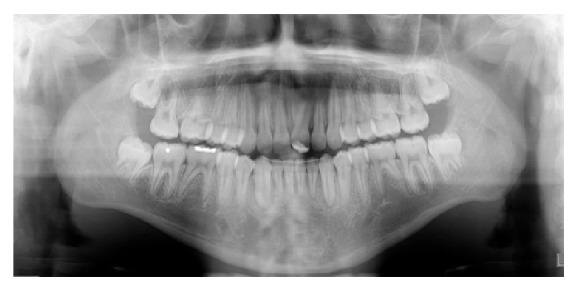
Stage 1: Orthodontic Treatment Final Panoramic Radiograph.

**Figure 12:**
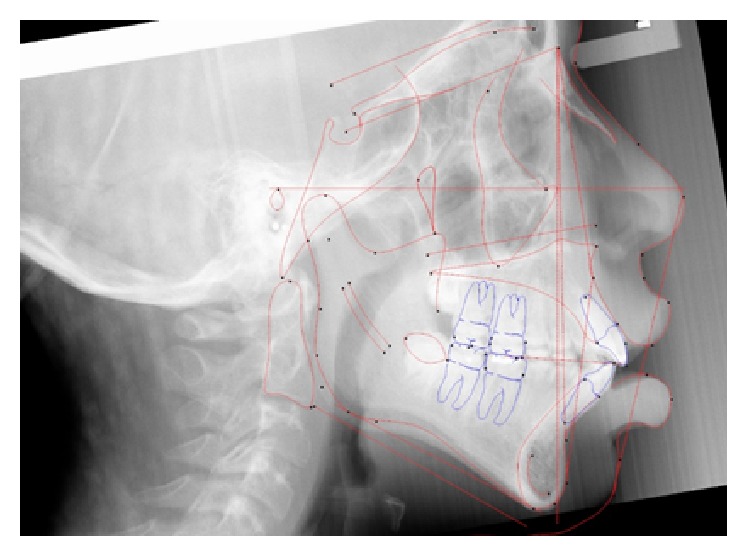
Stage 1: Orthodontic Treatment Cephalometric Radiograph.

**Figure 13:**
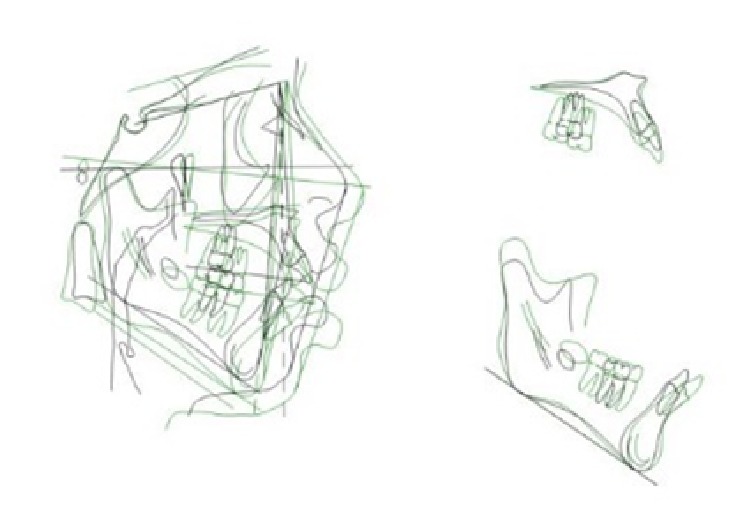
Stage 1: Orthodontic Treatment Superimposition.

Treatment time was 25 months and he was retained with a maxillary Hawley and fixed canine-to-canine mandibular retainer (Figures 9 [Fig fig10] [Fig fig11]–12). His final cephalometric analysis showed a Class II skeletal pattern with a retrognathic mandible and an ANB angle of 7.6°. He continued to have a convex skeletal and soft tissue profile with protrusive lips with a cervical development of CVS 4 at this time. The airway was widened in the two-dimensional view of the cephalometric radiograph when compared to his initial in Figure 5. Stage 1 treatment superimposition (Figure 13) showed there was bite opening with extrusion of upper molars, the lower incisors were flared, and the upper and lower lips moved forward 4 mm. and 6 mm., respectively.

Another sleep study was done on 2/11/13 to reevaluate the severity of his OSA following this initial stage of orthodontic treatment. Again sleep architecture was fragmented with arousals due to respiratory events. The microarousal index was 14, which was increased. Snoring was present throughout the study. This study documented no obstructive apneas, 19 central apneas, no mixed apneas and 33 hypopneas (of which 29 were associated with a 3% oxygen desaturation), and 18 respiratory effort related arousals (RERA) over the 504 minutes of recorded sleep. It showed his overall AHI to be 6.2 and the overall respiratory index (RDI includes RERAs) was 8.3. The non-REM RDI was 8.8 and the REM RDI was 5.5. The supine RDI was 57. The average oxyhemoglobin saturation was 96% while awake, 97% during REM, and 95% during non-REM sleep. The overall oxygen desaturation index during sleep was 6. The lowest oxygen saturation during sleep was 85%. The oxygen saturation was below 90% for less than 1% of the total sleep time. The EEG documented normal sinus rhythm. His average heart rate was 86 bpm and periodic limb movements were not observed. While much improved from his first sleep study it was felt that the additional surgical treatment would improve his moderate residual OSA. The University of Chicago Sleep Center had recommended nasal CPAP for the patient but he did not tolerate it well after several attempts. The patient was referred to the University of Illinois Oral Surgery Department for extraction of all four third molars and a possible advancement genioplasty to improve his airway.

**Figure 14:**
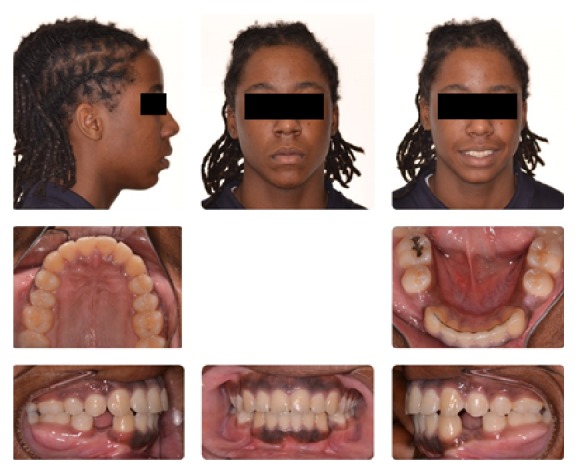
Stage 2: Orthodontic Treatment Initial Composite.

**Figure 15:**
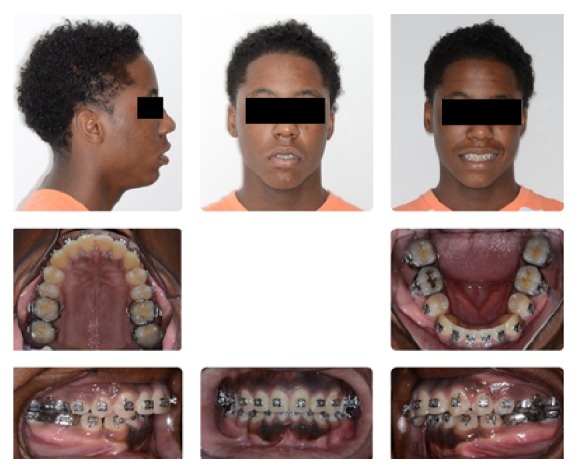
Stage 2: Presurgical Composite.

**Figure 16:**
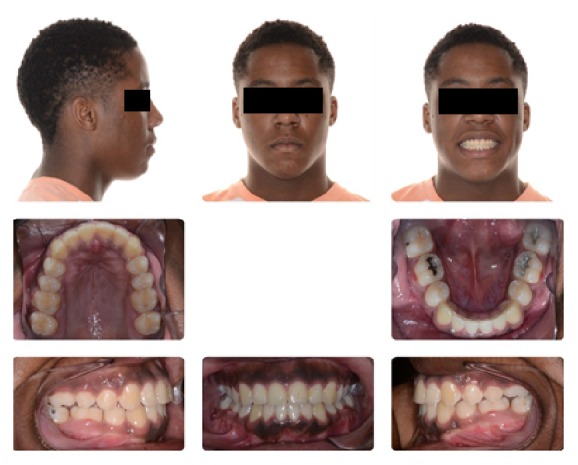
Stage 2: Orthodontic Treatment Final Composite.

**Figure 17 fig17:**
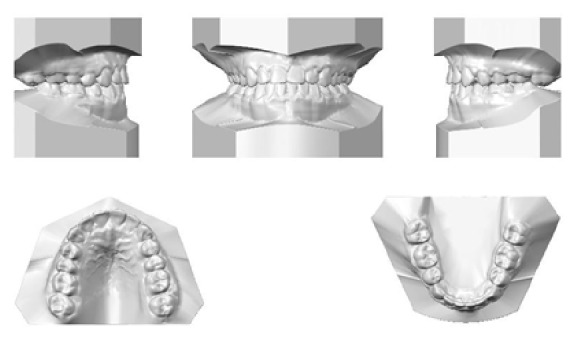
Stage 2: Orthodontic Treatment Final Models.

**Figure 18 fig18:**
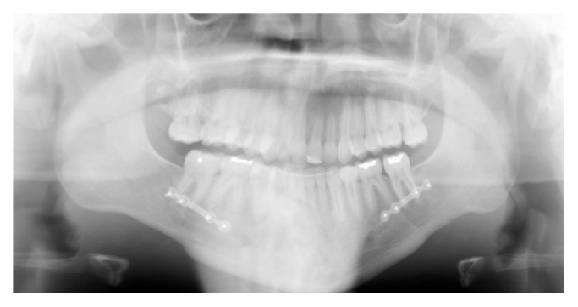
Stage 2: Orthodontic Treatment Final Panoramic Radiograph.

**Figure 19:**
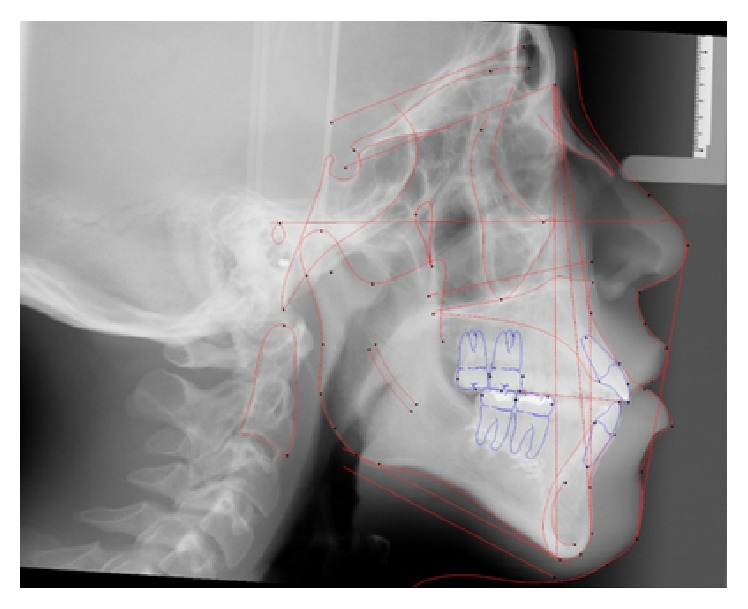
Stage 2: Orthodontic Treatment Final Cephalometric Radiograph.

**Figure 20:**
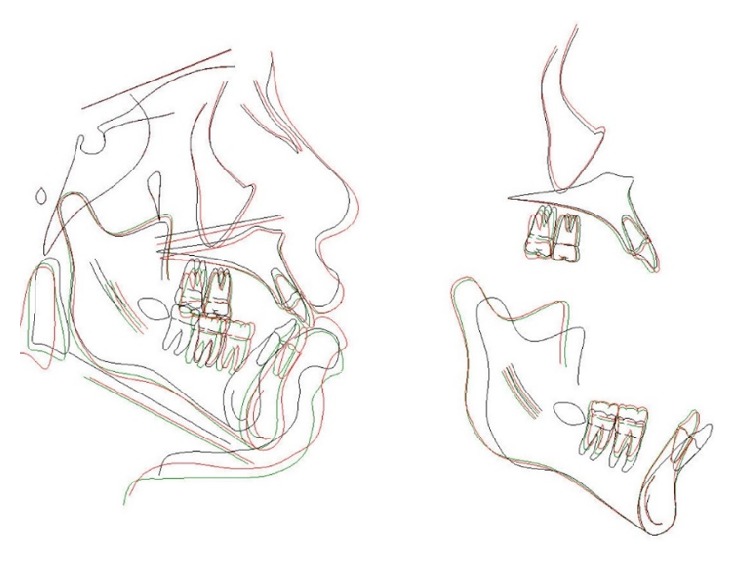
Overall superimposition, Stage 1 final (black line), presurgical (green line), and Stage 2 final (red line).

After evaluation by the Oral Surgery Department, it was felt that mandibular advancement surgery would more predictably lead to a correction of the patient's residual OSA due to his retrognathic mandibular position. At the treatment consult it was explained to the patient and his mother that he would need to go through another stage of orthodontic treatment to decompensate his dentition to maximize the anterior surgical movement of his mandible. It was agreed to extract his four third molars and both his lower first premolars and retract the lower anterior teeth so a maximum advancement of his mandible with a bilateral sagittal split osteotomy (BSSO) could be obtained (Figures 14 and 15). The patient was finished with Class I canines and Class III molars, and ideal overbite and overjet. This was accomplished in 19 months. He was retained with an upper Hawley and lower fixed canine-to canine retainer with an overlay Hawley at night (Figures 16 [Fig fig17] [Fig fig18]–19). Stage 2 final cephalometric radiograph (Figure 19) showed a marked increase in the size of his airway in this two-dimensional view. The overall superimposition (Figure 20) showed significant growth and anterior positioning of the mandible. The patient was referred for a follow-up sleep study at the time of final debonding. Again sleep architecture was fragmented with arousals due to respiratory events. The microarousal index was 11, which was increased. This study documented no obstructive sleep apneas, 5 central apneas, 1 mixed apnea, and 22 hypopneas (of which 15 were associated with 3% oxygen desaturation) over 367 minutes of recorded sleep. His overall AHI was 4.6 which is considered very mild. The overall RDI which includes RERAs was 4.6. The non-REM RDI was 5.5 and the REM RDI was 0.8. The supine RDI was 5.9. The average oxyhemoglobin saturation was 97% while awake, 98% during REM, and 96% during non-REM sleep. The overall oxygen desaturation index during sleep was 2. The lowest oxygen saturation during sleep was 90%. The EEG documented normal sinus rhythm. His average heart rate was 78 bpm and no periodic limb movements were observed. His mother also reported that there was a significant reduction in the patient's snoring. The patient was scheduled for continued retention checks in the University of Illinois Orthodontic Department and medical monitoring of his OSA with the Pediatric Sleep Disorders Program at the University of Chicago for Obstructive Sleep Apnea.

## 3. Discussion

Although adenotonsillectomy is considered a first line of treatment in pediatric OSA, the Pediatric Sleep Disorders Program at the University of Chicago felt that the patient was not a candidate for these procedures. Rather he was referred to the University of Illinois Orthodontic Department for evaluation and treatment. After evaluation it was felt that expansion of both upper and lower arches would be indicated to decrease his OSA symptoms with the possibility of orthognathic surgery if sufficient reduction in his AHI was not achieved. This is what occurred in the patient's treatment and necessitated his two stages of orthodontic treatment. It has been reported that an anteriorly titrated mandibular position reduced Obstructive Sleep Apnea severity, enlarged the velopharynx, and diminished the curvature of the anterior velopharyngeal wall. It is proposed that this change in the upper airway curvature associated with mandibular advancement may affect Obstructive Sleep Apnea severity through its effect on airflow dynamics [26]. Surgical maxillomandibular advancement is prescribed as a functional and curative treatment for OSA. It can result in significant improvement in the quality of life and reduction in OSA health-related risks [27–33].

Oral mandibular advancement appliances have been demonstrated to be effective in the treatment of mild to moderate OSA [34, 35]. They can also be used in severe OSA cases where the patient does not respond to CPAP therapy and does not want surgical treatment. Treatment with oral removable functional appliances, as the Twin Block appliance, has also been advocated to help growing pediatric OSA patients with a stimulation of mandibular growth [36, 37]. It was felt that this patient with severe OSA could be most predictably treated with a surgical approach as he did not tolerate CPAP treatment.

If left untreated, pediatric OSA can lead to many negative consequences that affect multiple target organs and systems. OSA can lead to behavioral disturbances, learning deficits, cardiovascular problems, compromised somatic growth, and decreased quality of life, depression, enuresis, and increased healthcare related costs [1]. Adults with untreated OSA have higher healthcare use [38]. Children with OSA are also heavy users of healthcare resources [39]. This often starts from the first year of life. Increased sickness in children with OSA is often related to lower respiratory diseases. In one study, the total number of hospital visits was 40% higher in children with OSA and these children needed 20% more repeated hospital visits compared to matched controls without OSA. A significant increase in referrals to ENT physicians and increased use of drugs were found in children with OSA [40]. Early diagnosis of OSA in childhood and adolescence is critical in reversing or eliminating the many negative health consequences of OSA in adulthood.

## 4. Conclusion

This case demonstrates the effectiveness of comprehensive orthodontic treatment combined with orthognathic surgery in the correction of malocclusion and reduction in the sequelae of Obstructive Sleep Apnea. There was significant improvement in the patient's sleep continuity and architecture with the elimination of obstructive apneas. Early diagnosis of OSA in childhood and adolescence is critical in reversing or eliminating the many negative health consequences of OSA in adulthood.
